# DYING WITH DEMENTIA IN NURSING HOMES: A POPULATION-BASED PERSPECTIVE OF DECEDENTS AND THEIR FAMILIES

**DOI:** 10.1093/geroni/igad104.1709

**Published:** 2023-12-21

**Authors:** Caroline Stephens, Rebecca Goodwin, Eli Iacob, Michael Hollingshaus, Ken Smith, Djin Tay, Rebecca Utz, Katherine Ornstein

**Affiliations:** University of Utah, Salt Lake City, Utah, United States; National Library of Medicine, NIH & University of Utah College of Nursing, Salt Lake City, Utah, United States; University of Utah, Salt Lake City, Utah, United States; University of Utah, Salt Lake City, Utah, United States; University of Utah, Salt Lake City, Utah, United States; University of Utah, Salt Lake City, Utah, United States; University of Utah, Salt Lake City, Utah, United States; Johns Hopkins University, Baltimore, Maryland, United States

## Abstract

Families play a critical role in person-centered end-of-life (EOL) care for nursing home (NH) residents with dementia. Many NH residents, however, do not have any family to advocate for care, which may contribute to poorer EOL outcomes. Little is known about the characteristics of families of persons with dementia who die in a NH despite the importance of family at EOL. This study aimed to describe the size and composition of first-degree families (FDF) of Utah NH residents with dementia who died 1998-2016 (n=18,842). Using the Utah Population Caregiving Database, we linked NH decedents with dementia to their FDF (n=54,174; spouses=11.3%; children=57.6%; siblings=30.7%) and compared characteristics of those with and without FDF. Compared to NH decedents with FDF (78.5%), those without (21.5%) were more likely to be older (median 87.3 vs 85.9), female (64.7% vs 57.1%), non-white/Hispanic (10.3% vs 3.4%), less educated (<9th grade; 42.2% vs 33.4%); die in a rural/frontier NH (24.8% vs. 23.2%); and less likely to have 2+ hospitalizations in the last 6 months of life (14.3% vs. 16.5%, all p<.001). Findings highlight social determinants of health that may increase the risk of poor EOL outcomes in an already vulnerable population. Understanding the nature of FDF relationship type on EOL outcomes for NH residents with dementia is an important next step in clarifying the role of families and understanding the supports they may need.

